# Anticancer Applications of Nanostructured Silica-Based Materials Functionalized with Titanocene Derivatives: Induction of Cell Death Mechanism through TNFR1 Modulation

**DOI:** 10.3390/ma11020224

**Published:** 2018-01-31

**Authors:** Santiago Gómez-Ruiz, Alberto García-Peñas, Sanjiv Prashar, Antonio Rodríguez-Diéguez, Eva Fischer-Fodor

**Affiliations:** 1 Departamento de Biología y Geología, Física y Química Inorgánica, ESCET, Universidad Rey Juan Carlos, Calle Tulipán s/n, E-28933 Móstoles (Madrid), Spain; sanjiv.prashar@urjc.es; 2 College of Materials Science and Engineering, Shenzhen Key Laboratory of Polymer Science and Technology, Guangdong Research Center for Interfacial Engineering of Functional Materials, Nanshan District Key Laboratory for Biopolymers and Safety Evaluation, Shenzhen University, Shenzhen 518060, China; alberto@szu.edu.cn; 3 Key Laboratory of Optoelectronic Devices and Systems of Ministry of Education and Guangdong Province, College of Optoelectronic Engineering, Shenzhen University, Shenzhen 518060, China; 4 Departamento de Química Inorgánica, Universidad de Granada, Facultad de Ciencias, Campus de Fuentenueva, Avda. Fuentenueva s/n, E-18071 Granada, Spain; antonio5@ugr.es; 5 Medfuture-Research Center for Advanced Medicine, Iuliu Hatieganu University of Medicine and Pharmacy, RO-400337 Cluj-Napoca, Romania; 6 Tumor Biology Department, The Oncology Institute “I. Chiricuta”, RO-400015 Cluj-Napoca, Romania

**Keywords:** nanostructured silica, titanocene, cytotoxicity, anticancer, tumor necrosis factor, TNFR1 modulation

## Abstract

A series of cytotoxic titanocene derivatives have been immobilized onto nanostructured silica-based materials using two different synthetic routes, namely, (i) a simple grafting protocol via protonolysis of the Ti–Cl bond; and (ii) a tethering method by elimination of ethanol using triethoxysilyl moieties of thiolato ligands attached to titanium. The resulting nanostructured systems have been characterized by different techniques such as XRD, XRF, DR-UV, BET, SEM, and TEM, observing the incorporation of the titanocene derivatives onto the nanostructured silica and slight changes in the textural features of the materials after functionalization with the metallodrugs. A complete biological study has been carried out using the synthesized materials exhibiting moderate cytotoxicity in vitro against three human hepatic carcinoma (HepG2, SK-Hep-1, Hep3B) and three human colon carcinomas (DLD-1, HT-29, COLO320) and very low cytotoxicity against normal cell lines. In addition, the cells’ metabolic activity was modified by a 24-h exposure in a dose-dependent manner. Despite not having a significant effect on TNFα or the proinflammatory interleukin 1α secretion, the materials strongly modulated tumor necrosis factor (TNF) signaling, even at sub-cytotoxic concentrations. This is achieved mainly by upregulation of the TNFR1 receptor production, something which has not previously been observed for these systems.

## 1. Introduction

Since the discovery of cisplatin (cis-diamminedichloridoplatinum (II)) by Rosenberg and coworkers [1], a wide variety of metal complexes for medicinal applications, such as diagnosis or therapy, have been designed [2,3]. The expansion in this area has mainly been motivated by the recurrent problems that metallodrugs present in cancer therapy, notably, the high number of side effects [4], low stability of the metal-based drugs in biological media [5], and the development of resistance by the cells treated with these compounds [6]. Thus, the search for novel chemotherapeutic metal-based drugs has been focused on complexes with central atoms, such as Fe, Ru, Ga, Au, Sn, and Ti, which have received wide attention as plausible alternatives to the use of platinum metallodrugs in cancer treatment [2]. 

In most of the studies of metal-based drugs in anticancer therapy, the cytotoxicity is normally correlated with the structure of the complex. However, in many cases, metallodrugs change their structure in the previous steps of passive or active transportation to the cell and membrane crossing, leading to a transformation of the coordination sphere of the metal and/or decomposition of the complex which makes them act more as a prodrug than, specifically, as a drug [7]. In this context, the use of encapsulators or carrier vehicles for the protection of the actives species and subsequent delivery to cancer cells is an approach of current interest for the scientific community [8,9]. A wide variety of nanostructured systems have been used to encapsulate metallodrugs and have been tested in anticancer chemotherapy both in vitro and in vivo [8]. One of the most interesting scaffolds for metallodrug-loading is nanostructured mesoporous silica which, loaded with titanium [9,10,11,12,13,14,15], tin [16,17], or other metallodrugs [8], has been shown to be highly effective against cancer cell lines.

The problems of metallodrugs associated with the development of multidrug resistance (MDR) are one of the main obstacles in the successful chemotherapeutic treatment of cancer [18]. It usually occurs with prolonged treatment of cancer and may lead to recurrence of the tumor and a decrease of the efficacy of other chemotherapeutic agents. Thus, it is very important to understand the biological targets of the metallodrugs and metallodrug-functionalized nanostructured materials in order to elucidate the mechanism of cell death promoted by these systems and how they are associated or not with MDR [18]. In this context, nanomedicinal strategies are currently being used to treat multidrug-resistant tumors [19]. 

In the search for novel more efficient alternative metallodrugs in cancer treatment, principally trying to overcome the problems associated with the use of metal-based drugs in chemotherapy, our group has reported pioneering work in the preparation of metallodrug-functionalized nanostructured materials based on nanostructured mesoporous silica functionalized with titanocene and organotin compounds which have been tested in vitro against several cancer cell lines [20]. Our previous studies have shown that these materials usually enhance the cytotoxic action of the metallodrug and act as “non-classical” drug-delivery systems, namely, they do not need the release of the metallodrug to be cytotoxic, acting as the entire nanoparticulated system [8]. We have observed that the metallodrug-functionalized silica-based nanostructured materials usually induce a higher metal prodrug uptake than their corresponding non-encapsulated metallodrug. This higher uptake leads to a greater cytotoxic activity and to a completely different mechanism of cell death [14]. In addition, we have observed that the dynamics of apoptotic morphological and functional changes is modified when the active titanium metallodrug species are incorporated into nanostructured silica-based systems as they induce the programmed cell death in tumor cell populations by impairing the damaged DNA repair mechanisms and by upregulation of intrinsic and extrinsic apoptotic signaling pathways [14]. Titanocene derivatives and titanocene-functionalized nanostructured systems have also proven to be active against various tumor cell lines, including cancers of the digestive system, by triggering apoptosis [14], the programmed cell death. From a mechanistic point of view, both the extrinsic and the intrinsic pathways of apoptosis can be targeted by titanocenes [21]. 

Looking for new mechanistic insights into metallodrug-functionalized nanostructured systems, one should bear in mind that the tumor necrosis factor alpha (TNF-α) has an important biologic function in apoptotic and inflammatory processes, exerted mainly by binding the tumor necrosis factor receptor 1 (TNFR1) [22]. In the extrinsic apoptotic pathways, the TNF-dependent mechanism requires an increased cell surface expression of TNFR1 [23], activation of mitogen activated protein kinases and caspases. TNFR1 activates the apoptotic signaling through nuclear factor-kappaB (NF-kappaB) and other mechanisms and induces cell death in colon tumor cells [24] and hepatic carcinoma [25]. Thus, more studies involving the tumor necrotic factors are needed to completely understand their role in cancer treatment.

In this context, we have prepared and characterized a series of cytotoxic titanocene derivatives which have been immobilized onto SBA-15 using two different synthetic protocols. The mesoporous silica-based material SBA-15 was chosen for its optimal properties as drug adsorbent [8] which are based on its textural properties which include large specific surface area, very uniform pore sizes (of around 6.5 nm), high textural porosity, high surface-to-volume ratio, and good thermal stability. The cytotoxic activity of these materials has been tested against three human hepatic carcinoma (HepG2, SK-Hep-1, Hep3B) and three human colon carcinoma (DLD-1, HT-29, COLO320) cell lines in vitro. In addition, we have evaluated the in vitro antitumor activity of four titanocene-functionalized materials, analyzing their cytotoxicity, the cellular metabolic activity alteration, and the modulation of the proinflammatory chemokines. The mechanistic study shows that the cells metabolic activity was modified by a 24-h exposure in a dose-dependent manner. Furthermore, despite not having a significant effect on TNFα or the proinflammatory interleukin 1α secretion, the materials strongly modulated the tumor necrosis factor (TNF) signaling, even at sub-cytotoxic concentrations, mainly by upregulation of the TNFR1 receptor production. To the best of our knowledge, this is the first example reported in the literature of upregulation of TNFR1 production by titanium-functionalized nanostructured materials.

## 2. Results and Discussion

### 2.1. Synthesis and Characterization of Metallodrug-Functionalized Nanostructured Materials

**Scheme 1:**
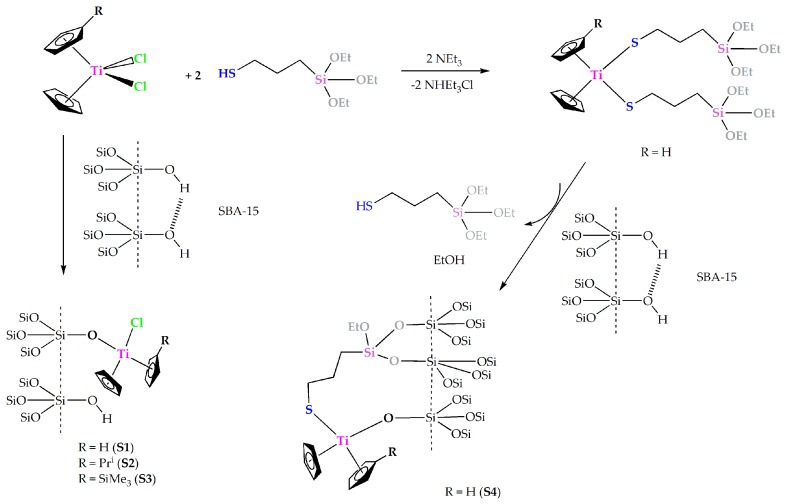
Synthesis of **S1**–**S4**.

A simple grafting reaction of three simple titanocene derivatives with different substituents, namely, [Ti(η^5^-C_5_H_5_)_2_Cl_2_] (**1**), [Ti(η^5^-C_5_H_5_)(η^5^-C_5_H_4_Pr^i^)Cl_2_] (**2**), and [Ti(η^5^-C_5_H_5_)(η^5^-C_5_H_4_SiMe_3_)Cl_2_] (**3**) on dehydrated (treated for 24 h at 150 °C under vacuum) SBA-15, has been carried out to give the corresponding materials SBA-15/[Ti(η^5^-C_5_H_5_)_2_Cl_2_] (**S1**), SBA-15/[Ti(η^5^-C_5_H_5_)(η^5^-C_5_H_4_Pr^i^)Cl_2_] (**S2**), and SBA-15/[Ti(η^5^-C_5_H_5_)(η^5^-C_5_H_4_SiMe_3_)Cl_2_] (**S3**) (Scheme 1). For the grafting of the compounds, two different functionalization reactions were used. The first consisted in the treatment of dehydroxylated SBA-15 with solutions of the corresponding titanocene complex in toluene at 110 °C. The Ti/SiO_2_ ratio of the reaction was always 7%. The second method is a tethering reaction of a titanocene derivative containing triethoxysilyl fragments [Ti(η^5^-C_5_H_5_)_2_{SCH_2_CH_2_CH_2_Si(OEt)_3_}_2_] (**4**) with SBA-15 to give the material SBA-15/[Ti(η^5^-C_5_H_5_)_2_{SCH_2_CH_2_CH_2_Si(OEt)_3_}_2_] (**S4**). The latter reaction proceeded by the elimination of ethanol and formation of the corresponding silylated material with formation of Si–O–Si bonds (Scheme 1). All materials **S1**–**S4** were characterized by various techniques and the results were compared with those of the starting material SBA-15.

#### 2.1.1. X-ray Fluorescence

The functionalization reactions were carried out using a theoretical 7% of wt % Ti (Ti/SiO_2_). The X-ray fluorescence data showed wt % Ti for functionalized materials **S1**–**S4** of 1.41%, 1.32%, 1.33%, and 6.47%, respectively (Table 1). In the case of materials **S1**–**S3** the functionalization reactions yielded a low incorporation of titanium in the material. This low functionalization rate is most probably because the initial dehydrated SBA-15 had a low number of hydroxyl groups due to its treatment under vacuum at high temperature, consequence of which there is only limited reaction between the chlorido ligands of the titanocene complexes with the silanol groups of the SBA-15. These reactions usually lead to *μ*-oxo surface species (Scheme 1) which are the most abundant functionalization species, however, a very small quantity of titanocene complexes may also be adsorbed inside the pores of SBA-15. This low titanocene-functionalization rate is in agreement with previous studies of our group on SBA-15 materials [10] which have shown a maximum loading of metallocene complexes on SBA-15, of ca. 2% of metal, even when starting from high Ti/SiO_2_ ratios of up to 5% [10]. This low functionalization was attributed both to the saturation of the surface and to the weak basicity of both chlorido ligands of the titanocene complexes and the Si–OH groups of SBA-15 [10].

**Figure 1:**
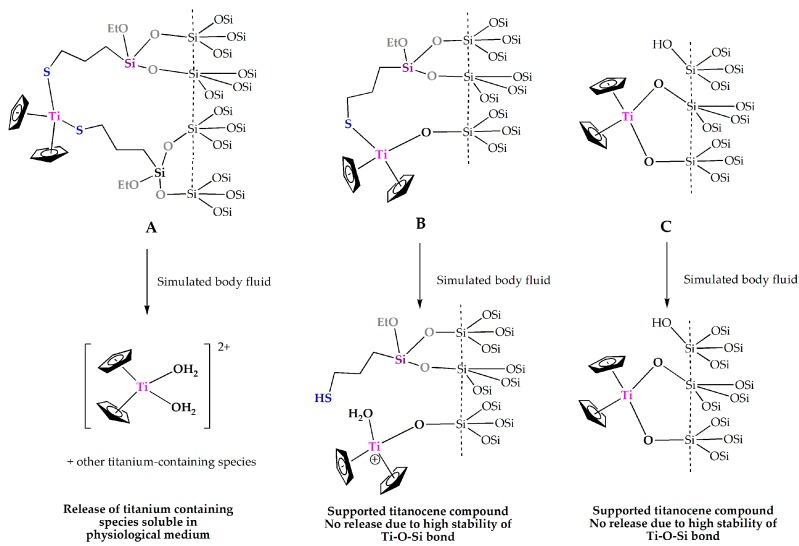
Functionalization species: (**A**) by the elimination of ethanol groups; (**B**) by the elimination of ethanol groups and a thiolate ligand, which leads to the formation of a Ti–O–Si bond; and (**C**) by the elimination of both thiolate ligands, which leads to the formation of two Ti–O–Si ligands. The figure also represents the proposed release species in simulated body fluid.

The reaction of [Ti(η^5^-C_5_H_5_)_2_{SCH_2_CH_2_CH_2_Si(OEt)_3_}_2_] (**4**) with SBA-15 led to the elimination of one of the 3-mercaptopropyltriethoxysilane ligands and ethanol. The functionalization of the materials was achieved by the binding of the triethoxysilyl groups to SBA-15 via formation of new Si–O–Si bonds. Nevertheless, formation of Ti–O–Si bonds due to the elimination of one of the thiolato ligands bound to the titanium center also occurred (Scheme 1). Thus, **S4** consists of a mixture of titanocene-functionalized species formed by “Cp_2_Ti(O-SBA-15)_2_” (Figure 1A), “Cp_2_Ti{SCH_2_CH_2_CH_2_Si(OEt)_2_O-SBA-15}(O-SBA-15)” (Figure 1B), and “Cp_2_Ti{SCH_2_CH_2_CH_2_Si(OEt)_2_O-SBA-15}_2_” species (Figure 1C). Elemental analysis data obtained by X-ray fluorescence showed a very good functionalization rate of **S4**, 6.47 wt % Ti (Table 1). It is clearly seen that the functionalization rate by using titanocene complexes with triethoxysilyl moieties is much higher and is in agreement with previous reports from our group [12].

#### 2.1.2. Powder X-ray Diffraction

**Figure 2:**
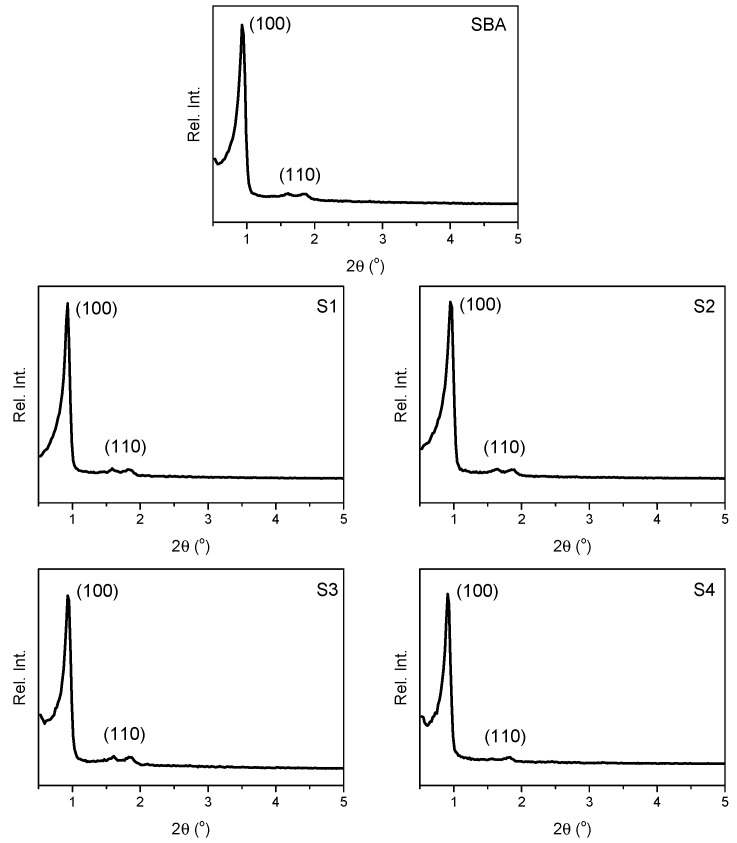
XRD diffraction patterns of SBA-15, **S1**–**S4**.

All the synthesized materials were characterized by XRD. Their diffractograms show typical reflections for hexagonally-ordered mesoporous materials. Unmodified SBA-15 showed a well-resolved pattern at low 2θ values with an intense (100) diffraction peak at 0.93° and another peak of lower intensity at 1.84°, assigned to the (110) Miller plane (Figure 2). This system can be indexed as a hexagonal lattice with d-spacing values of 73.6 and 14.77 Å, respectively (Table 2).

After functionalization with the titanocene derivatives, no notable changes were observed in the XRD pattern and position of the diffraction peaks (Figure 2). However, a clear decrease of intensity was observed due to the partial blocking of the dispersion centers of the material by the titanocene derivatives. In all cases, the diffraction pattern of **S1**–**S4** can be indexed as a hexagonal lattice with *d*-spacing values of ca. 73 Å (Table 2). These results suggest that the structural order of the synthesized material is maintained after functionalization with the titanocene derivatives.

#### 2.1.3. N_2_ Adsorption-Desorption Isotherms

**Figure 3:**
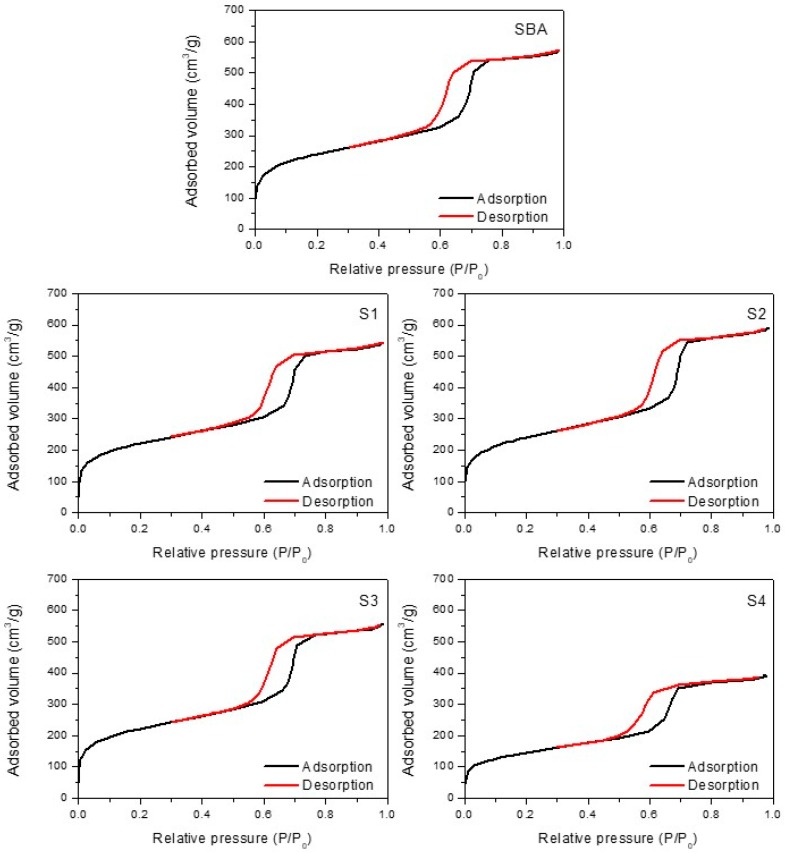
Nitrogen adsorption/desorption isotherms of SBA-15, **S1**–**S4**.

All the synthesized materials **S1**–**S4** were also characterized by nitrogen adsorption/desorption isotherms, observing the formation of type IV isotherms (according to the IUPAC classification [26,27]) which have an H2b hysteresis loop corresponding to a typical mesoporous material (Figure 3). The complete adsorption data are summarized in Table 3.

The isotherms show that the BET surface area (S_BET_) of unmodified SBA-15 is ca. 860 m^2^/g and after functionalization with the titanocene derivatives (**S1**–**S4**) slightly decreases. Capillary condensation of nitrogen within the uniform mesoporous structure was observed in all materials at a relative pressure (P/P_0_) of ca. 0.4, although the inflection position shifted slightly toward lower relative pressures after functionalization. In addition, BJH average pore diameter decreases for **S1**–**S4** compared with that of unmodified SBA-15 while the wall thickness significantly increases. This phenomenon confirms a reduction in pore size in functionalized materials which is probably because the titanocene derivatives are located inside the pore of the mesoporous system.

When analyzing the pore size distributions of the all the synthesized materials, a homogeneous narrow distribution is observed (see Figure S1 of supporting information), indicating the high degree of homogeneity of the materials. Thus, taking into account all the adsorption data, one can envisage that the titanium complexes loaded in the mesoporous materials are located in the pores of the system.

#### 2.1.4. Solid-State NMR Spectroscopy

**Figure 4:**
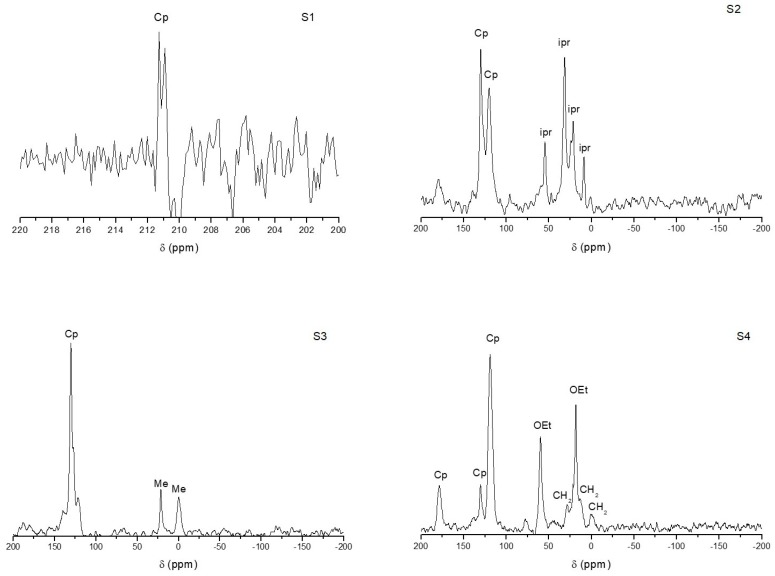
^13^C CP MAS NMR spectra of SBA-15, **S1**–**S4**.

The ^13^C CP MAS spectra of materials **S1**–**S4** (Figure 4) have been recorded and showed the signals of the different carbon atoms of the supported titanocene compounds. Specifically, the spectrum of **S1** shows a set of two broad signals at ca. 210 ppm corresponding to the Cp ligands of supported [Ti(η^5^-C_5_H_5_)_2_Cl_2_]. The spectrum of **S2** shows two sets of signals, one composed of four signals between 0 and 55 ppm which corresponds to the aliphatic carbon atoms of the isopropyl group of supported [Ti(η^5^-C_5_H_5_)(η^5^-C_5_H_4_Pr^i^)Cl_2_] and the second made up of two signals at ca. 120–130 ppm assigned to the carbon atoms of the cyclopentadienyl rings. The spectrum of **S3** also shows two groups of signals for the different carbon atoms of the supported complex [Ti(η^5^-C_5_H_5_)(η^5^-C_5_H_4_SiMe_3_)Cl_2_] (**3**); firstly, two signals between 0 and 25 ppm corresponding to the carbon atoms of the methyl groups of SiMe_3_ and, secondly, a group of signals between 100 and 150 ppm due to the carbon atoms of the cyclopentadienyl groups. Finally, the spectrum of material **S4** showed three signals of low intensity between 0 and 30 ppm assigned to the three carbon atoms of the alkylic chain of the thiolato ligand, two broad signals at ca. 20 and 60 ppm corresponding to the carbon atoms of the ethoxy fragments and a set of three signals between 100–200 ppm due to the carbon atoms of the cyclopentadienyl ligands of the titanocene derivative [Ti(η^5^-C_5_H_5_)_2_{SCH_2_CH_2_CH_2_Si(OEt)_3_}_2_].

**Figure 5:**
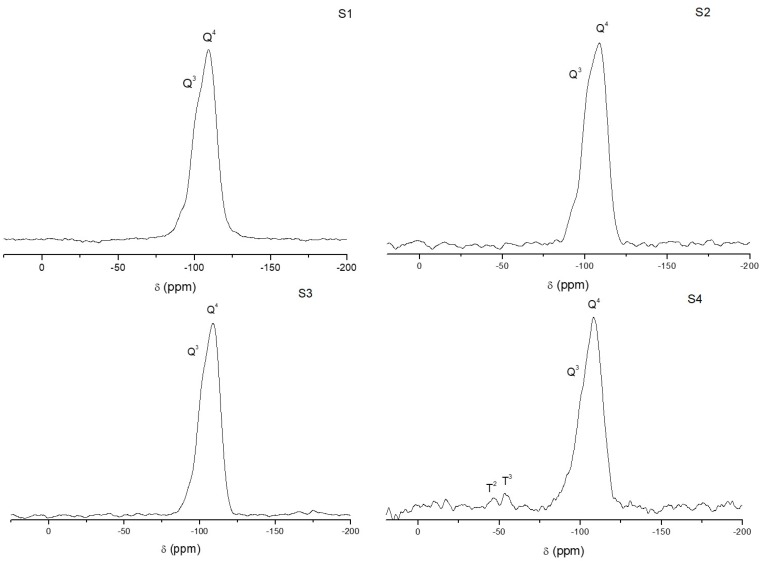
^29^Si MAS NMR spectra of SBA-15, **S1**–**S4**.

^29^Si MAS NMR spectra of SBA-15 and materials **S1**–**S4** (Figure 5) were also recorded. The spectrum of the non-functionalized SBA-15 shows the signals corresponding to the silicon atoms with hydroxyl bound groups [Si(OSi)_2_(OH)_2_] (Q_2_, at −91.5 ppm), the signal of [Si(OSi)_2_(OH)] (Q_3_, at −100.6 ppm) and the resonance of [Si(OSi)_2_] (Q_4_, at −110 ppm). After incorporation of the titanium-based metallodrugs, slight modifications of the intensity of Q_3_ and Q_2_ signals were observed which is a consequence of the functionalization by the titanocene compounds as previously reported in other studies [10,11,12]. It is important to note, that, although material **S3** is functionalized with a titanocene complex with a trimethylsilyl group, the signal of the silicon atoms of this group was not observed, probably because of the low degree of functionalization and the high number of silicon atoms of the silica matrix (SBA-15) which obscure the appearance of the resonance of the silicon atom of SiMe_3_. This phenomenon was previously observed by us in other studies [11]. Interestingly, the spectrum of **S4** showed, in addition to the Q_2_, Q_3_, and Q_4_ signals, two new peaks of low intensity were recorded at approximately −48 and −55 ppm which have been assigned to T_2_ ((SiO)_2_SiOH–R) and T_3_ ((SiO)_3_Si–R) sites of silica, respectively [10,11,12].

#### 2.1.5. Thermogravimetry

All the synthesized materials SBA-15 and **S1**–**S4** were characterized by thermogravimetric studies (see Figure S2 of supplementary material). The TGA curves show an initial loss of mass of approximately 5% due to adsorbed water on the SBA-15. In addition, in the titanocene-functionalized materials (**S1**–**S4**) a second loss of mass is observed between 250 and 550 °C which is due to the degradation process of the grafted titanocene derivative (exothermic process). The weight losses are of about 5–7% for materials **S1**–**S3** while, in **S4**, this is much higher (ca. 15%) and confirms the higher functionalization rate of this material compared with **S1**–**S3**.

#### 2.1.6. IR and UV Spectroscopy

UV-VIS spectra of the synthesized materials **S1**–**S4** were recorded and compared with that of unmodified SBA-15. The latter shows a broad peak at 250 nm while functionalized materials **S1**–**S4** show a shoulder between 290 and 370 nm which is due to the incorporation of the different titanocene derivatives (see Figure S3 of supplementary materials).

In addition, the FTIR spectrum of SBA-15 shows several characteristic bands; a broad band between ca. 3450 and 3200 cm^−1^, which was assigned to O–H stretching of the silanol groups of the material and the adsorbed water molecules, a broad strong band at ca. 1100 cm^−1^ due to the siloxane (Si–O–Si) groups, a stretching band at ca. 900 cm^−1^ of the Si–O bonds of the silanol groups, and an additional band at ca. 1630 cm^−1^ assigned to the deformation vibrations of adsorbed water molecules. Materials **S1**–**S4** presented weak intensity bands for aliphatic and aromatic C–H stretching vibrations between 3000 and 2800 cm^−1^ and a shoulder at ca. 1550 assigned to the vibrations of the cyclopentadienyl ligands (see Figure S4 of supplementary materials).

#### 2.1.7. SEM and TEM

**Figure 6:**
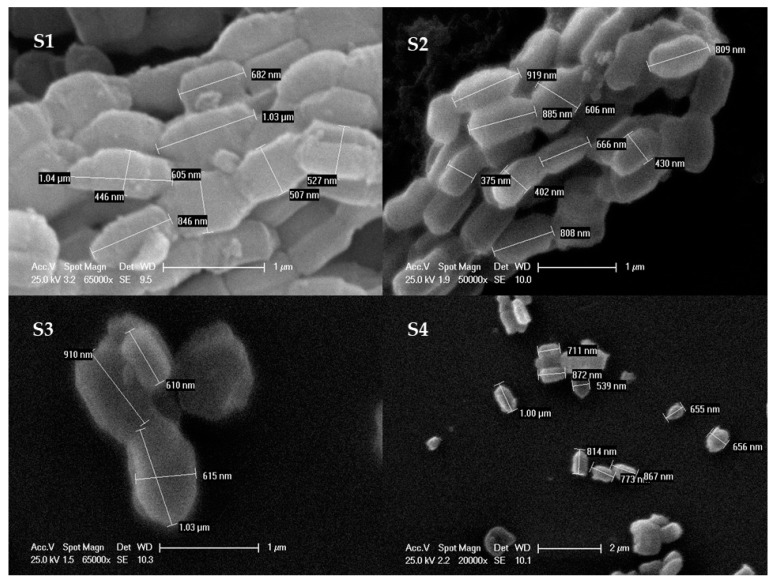
SEM images of **S1**–**S4.**

All the synthesized materials have been characterized by scanning electronic microscopy (SEM), in order to determine the morphology of the nanostructured systems. According to the micrographs, there were no significant differences between the non-functionalized SBA-15 and the titanocene-functionalized materials **S1**–**S4**. The unmodified SBA-15 shows uniform morphology of nanostructured rods (with narrow distribution of particle size of ca. 850 nm long and 400 nm width). The SEM images also show that after functionalization with the titanocene derivatives and formation of materials **S1**–**S4** (Figure 6), morphology and particle size do not change significantly, indicating that the functionalization step does not influence the particle shape and size.

**Figure 7:**
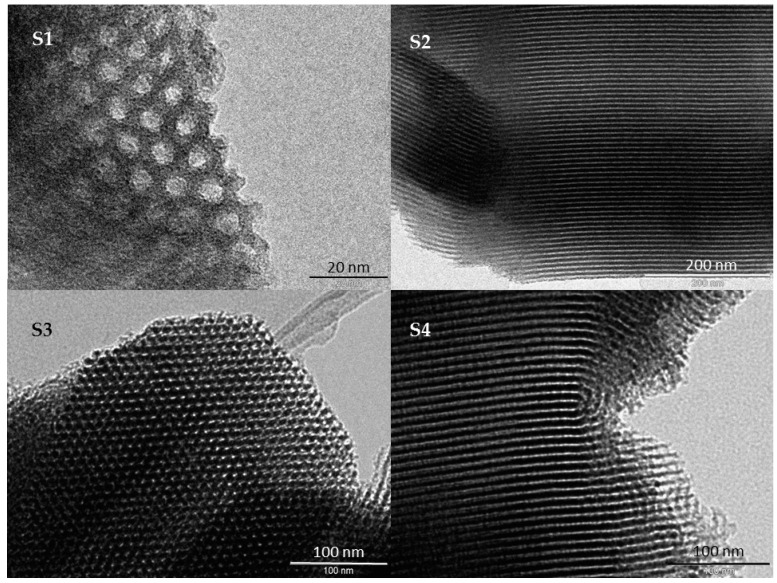
TEM images of **S1**–**S4.**

In addition, functionalized materials **S1**–**S4** have been characterized by transmission electronic microscopy (TEM). Figure 7 shows the transmission electron micrograph (TEM) images of SBA-15 functionalized materials **S1**–**S4** which present a highly-ordered structure with hexagonally-ordered porous parallel channels.

### 2.2. Qualitative Study of the Interactions with DNA

**Figure 8:**
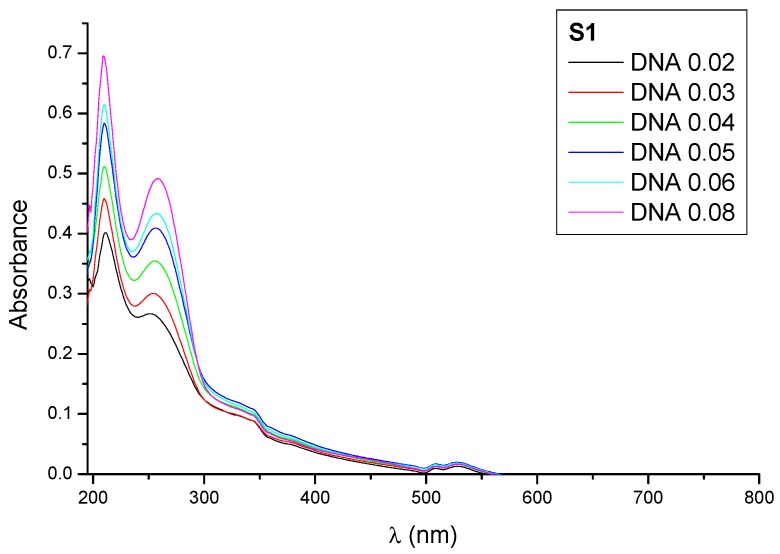
Binding study of **S1** upon increasing concentrations of DNA.

It is well-known from the accepted mechanism of action of titanocene derivatives that DNA is one of the possible biological targets of materials **S1**–**S4** in their cellular action [21]. In this context, the absorption spectra of material **S1** in the presence of various increasing concentrations of DNA has been recorded as a model simulating the behavior of these systems with DNA (Figure 8). 

Figure 8 shows that increasing concentrations of DNA leads to increases of the absorbance of the suspensions and a very slight blue shift of the peaks (hypsochromic effect) which indicate a possible adsorption of the DNA on the surface of the studied particles forming ground state adducts DNA-particle as reported previously by us [13] and another group [28]. Thus, electrostatic interactions between **S1** and DNA are proposed to be responsible for the adsorption of DNA.

### 2.3. Cell Growth Inhibition

The cytotoxicity of the materials was evaluated on three hepatic and three colorectal cell lines. Among hepatic cell lines, in HepG2 no genetic mutation has yet been identified; the Hep3B hepatocellular carcinoma is positive for the presence of Hepatitis B viral DNA and SK-Hep1 is metastatic, BRAF- and cyclin-dependent kinase inhibitor 2A (CDKN2A)-mutant cell line. In colon cancer cell lines: HT-29 is derived from a primary colon carcinoma, bearing several mutations: on BRAF (member of RAS family), Adenomatous polyposis coli (APC), phosphatidylinositol-4,5-bisphosphate 3-kinase (PIK3CA), SMAD4 and TP53 genes; Colo320 has APC and TP53 mutations; DLD-1 cells are APC-, TP53-, PIK3CA-, and K-RAS mutant. The genetic burden of the chosen cell lines made them an appropriate model for the in vitro testing of the materials. The colorimetric MTT cytotoxicity testing revealed a moderate inhibitory potential of the compounds after 24 h of treatment (Table 4). 

Our cytotoxic study shows that even though the Ti functionalization rate of the materials is relatively low, the cytotoxic nature of the hybrid materials is significant. These results are in agreement with previous studies in the field carried out by our research group [10,11,12,13,14,15,16,17]. 

Thus, in all cancer cell lines, **S3** and **S4** showed a superior cytotoxicity than **S1** and **S2** (one-way ANOVA, Bonferroni post-test, in the 95% confidence interval). Surprisingly, in Hep2G cells (which do not bear mutations), the growth inhibition was the weakest among all cell lines with the EC_50_ values being quite large, above the limit where the compounds can be considered as cytotoxic. Even so, the activity of **S4** was significantly higher than all the other compounds. In Hep3B, SK-Hep-1, DLD-1 the activity of **S3** and **S4** was comparable and significantly different from **S1** and **S2**. In HT-29 cells the cytotoxicity of **S4** was higher than that of **S3**; in COLO320 cells the inhibition was weak and in this cell line **S3** showed the highest activity. According to these results, the cytotoxic activity found for materials **S1**–**S4** are due to the particle action and not to the release of titanocene compounds or the non-functionalized materials (unmodified SBA-15 particles), as they both have been previously tested against similar cancer cell lines showing negligible cytotoxic activity [10,11,13].

An additional study against normal human cells LIV (normal human hepatic cell line) and BJ (CRL-2522, normal skin fibroblast cell line) was carried out as a model of non-malignant human cells, in order to determine the potential selectivity of the cytotoxic action of the synthesized materials on cancer cell lines. Previous studies showed the analogy between tumor colon cells and BJ fibroblasts toxicity [29]. Thus, due to their high proliferation rate, BJ cells are usually susceptible to toxic compounds and very sensitive to extrinsic and intrinsic stressors constituting a valuable standard for testing the toxicity of therapeutic agents in vitro when colon cancer is in study. In addition, several reports have already highlighted the importance of the skin fibroblast model in the determination of the selectivity of therapeutic agents [30,31]. The toxicity study carried out using the selected normal human cell lines for materials **S1**–**S4** showed that their inhibitory effect is below those observed for the tumor cells (higher EC_50_ values), without exception. The cytotoxicity of **S1**–**S4** was compared with that of the antitumor drug oxaliplatin, widely used in colorectal and liver pathology [32]. The antitumor action of oxaliplatin was generally better than that of **S1**–**S3** titanocene-loaded nanostructures, except against the highly proliferative HT-29 cells, with multiple mutations, where all titanocene-based materials are more active than the standard cytotoxic drug. Moreover, **S4** inhibitory activity was significantly higher than that of oxaliplatin (Table 4) in Hep3B hepatic tumor cells and DLD-1 colon carcinoma. Furthermore, the ratio of the normal cells EC_50_ to tumor cells EC_50_, or the in vitro selectivity of **S1**–**S4** is above 1 for each material. The largest ratios were calculated for **S4** and **S3**, being much higher than those of oxaliplatin showing, therefore, a higher selectivity for the titanocene-functionalized materials.

### 2.4. Effect on Intracellular Metabolic Activity

The intracellular reducing potential of tumor populations was evaluated using Alamar Blue fluorescent staining. The same concentrations were used as in the viability testing. In all cell lines, the tendency was a decrease in metabolic rate following the treatment with **S1**–**S4** (Table 5). For material **S4** the reducing effect was evident in all cell lines except the HepG2 cell line. **S2** was able to inhibit the metabolic activity of four cell lines, while **S1** inhibited the metabolic activity only in three.

### 2.5. Effect on Inflammatory Processes

The action of **S1**–**S4** caused no significant changes in interleukin-1α (IL-1α) concentrations in treated cells’ supernatants.

**Figure 9:**
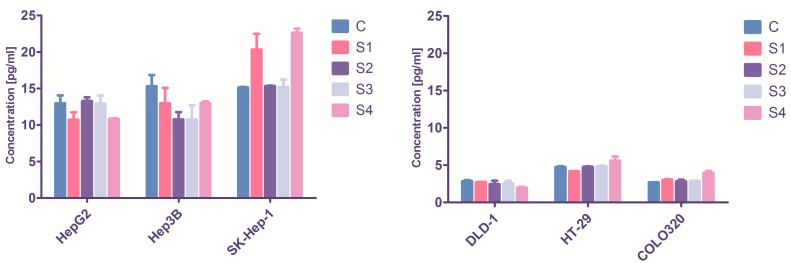
Quantitative evaluation of soluble IL-1α in hepatic HepG2, Hep3B, SK-Hep1, and colon DLD-1, HT-29 and COLO320 tumor cells, at the EC_50_ concentration of titanocene-functionalized materials **S1**–**S4**.

Our study confirmed that the hepatic cells are IL-1 secretor cell lines [34], and their basal IL-1α concentration was significantly higher those of colon carcinoma cells, which normally need IL-1α addition to be guided towards immune activation (Figure 9). Nevertheless, previous studies indicated that the cancer cells directly produce the proinflammatory IL-1α [35], a molecule with a dual role in tumor progression. Among hepatic cell lines, only in SK-Hep-1 was there a significant increase in IL-1α concentration following **S1** and **S4** treatment (two-way analysis of variance, *p* < 0.05). In colon carcinoma, **S4** significantly inhibited IL-1α in DLD-1 cells, while in HT-29 cell line an overexpression was observed. **S1**–**S3** influence on IL-1α was not considerable.

### 2.6. TNFR1

**Figure 10:**
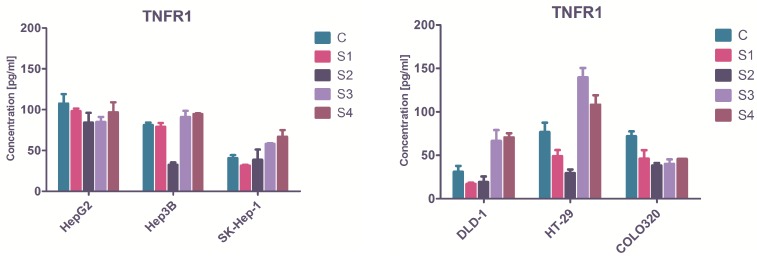
The concentration of tumor necrosis factor receptor 1 (TNFR1) in hepatic and colon cancer cell lines, after in vitro treatment with **S1**–**S4** at their EC_50_ concentrations.

Another parameter of interest for the cell growth inhibitory effect is the tumor necrosis factor alpha (TNF-α). Following its quantitative evaluation in the cell culture supernatants, the statistical analysis does not indicate substantial differences between the treated and untreated cells TNF-α concentration, or among the different materials analyzed (data not shown). Therefore, the soluble form of TNF receptor1 (TNFR1) was assessed, since a recent study has highlighted the importance of this molecule in tumor cells death pathways [36]. TNFR1 displayed significant changes after the cells treatment with **S1**–**S4**, at EC_50_ concentrations for each cell line. The basal TNFR1 values in the cell media supernatants of untreated cells were, as expected, different in every population since they derived from different tumor types and they have distinct histology (Figure 10). After the 24-h in vitro treatment, **S1** displayed no significant effect on the soluble TNFR1 production. When the treatment with **S2** was applied, TNFR1 concentration decreased in HepB3, HT-29, and COLO320 cells (one-way analysis of variance, *p* < 0.05). Contrarily, **S3** augmented the TNFR1 production in one hepatic (SK-Hep1) and all colon cell lines (DLD-1, HT-29, COLO320). **S4** also increased the receptors concentration in two hepatic (Hep3B, SK-Hep1), and two colon cell lines (DLD-1, HT-29).

It is known that the extrinsic apoptotic cell death pathway is initiated by TNFR1 signaling [37], and the promotion of proapoptotic TNFR1 can suppress cancer cell growth [24] and the selective activation of TNFR1. No mathematical correlation was found between IL-1 and TNFR1 values. In all cell lines, where the **S3** and **S4** augmented the proapoptotic TNFR1 in a significant manner, the metabolic activity of the cells was significantly reduced (Table 5). Initial in vivo studies showed that titanocene derivatives are able to cause necrosis in tumor cells [38]. However, subsequent studies indicated other titanocene-driven cell death mechanisms based on apoptosis and paraptosis [21]. Bearing in mind that TNFR1 is an apoptosis inducer and it is also capable of initializing cell death by necroptosis through TNFR1 [39], nanostructured materials **S1**–**S4** are probably implicated in both apoptotic and necrotic cell death processes in the liver or colon cancer cell populations.

### 2.7. Study of the Titanium Release

In order to gain more insights on the action of titanocene-functionalized materials, release of titanium for **S1** was studied under simulated physiological conditions (pH = 7.4). Thus, simulated body fluid (pH = 7.4) was added to **S1** and the suspension was incubated at 37 °C in a water bath for 1, 6, 24, 48, and 96 h. The suspensions were then centrifuged and the remaining material was analyzed by X-ray fluorescence showing almost the same wt % Ti as that before the treatment under physiological conditions (wt % Ti of between 1.11 and 1.21%, Table 6). This indicates that the release of titanocene derivative in these experiments was minimal and not significant enough to be considered as the responsible phenomenon that causes the cytotoxic activity of the materials. Therefore, as observed previously, the cytotoxic activity of functionalized material **S1** is most probably due to the particle action and not to the release of the titanium metallodrug.

However, release studies carried out under the same conditions for material **S4** in simulated body fluid showed a substantial titanium release after 96 h of ca. 30% of the loaded titanium. As explained before, **S4** consists of a mixture of titanocene-functionalized moieties formed by “Cp_2_Ti(O-SBA-15)_2_” species (Figure 1A), “Cp_2_Ti{SCH_2_CH_2_CH_2_Si(OEt)_2_O-SBA-15}(O-SBA-15)” fragments (Figure 1B), and “Cp_2_Ti{SCH_2_CH_2_CH_2_Si(OEt)_2_O-SBA-15}_2_” species (Figure 1C) from which only species B does not have Ti–O–Si bonds but Ti–S bonds which can be hydrolyzed under physiological conditions to release titanium containing soluble species to the medium, as was actually observed in the release experiments (Table 6). Therefore, cytotoxic action of material **S4** may be due to a dual activity from the released soluble titanium-containing species and the particle action.

## 3. Materials and Methods 

### 3.1. General Conditions

All reactions were performed using standard Schlenk tube techniques in an atmosphere of dry nitrogen. Solvents were distilled from the appropriate drying agents and degassed before use. The reagents used in the preparation of the corresponding metallocene complexes, such as cyclopentadiene dimer, [TiCl_4_(THF)_2_], Na(C_5_H_4_Pr^i^), TEOS, and Pluronic 123, were purchased from Sigma Aldrich (Tres Cantos, Spain) and used directly without further purification. 3-Mercaptopropyltriethoxysilane was purchased from Fluorochem Ltd. (Derbyshire, UK) and used without further purification.

### 3.2. General Conditions on the Synthesis and Characterization of the Titanocene Complexes

Li{C_5_H_4_(SiMe_3_)}, [Ti(η^5^-C_5_H_5_)_2_Cl_2_] (**1**) and [Ti(η^5^-C_5_H_5_)Cl_3_] were prepared according to the procedures from the literature [40]. The previously-reported titanocene complexes [Ti(η^5^-C_5_H_5_)(η^5^-C_5_H_4_Pr^i^)Cl_2_] (**2**) and [Ti(η^5^-C_5_H_5_)(η^5^-C_5_H_4_SiMe_3_)Cl_2_] (**3**) were prepared by the reaction of [Ti(η^5^-C_5_H_5_)Cl_3_] and Na(C_5_H_4_Pr^i^), or Li{C_5_H_4_(SiMe_3_)} (1:1) in THF, respectively. For the synthesis of [Ti(η^5^-C_5_H_5_)_2_{SCH_2_CH_2_CH_2_Si(OEt)_3_}_2_] (**4**) a slight modification of our reported procedure [12] was carried out. A solution of 3-mercaptopropyltriethoxysilane (1.43 g, 6.02 mmol) in toluene (25 mL) was added dropwise over 15 min to a solution of [Ti(η^5^-C_5_H_5_)_2_Cl_2_] (0.75 g, 3.01 mmol) in toluene (15 mL) at room temperature. The reaction mixture was stirred for 2 h, subsequently NEt_3_ (0.87 mL, 6.02 mmol) was added dropwise. The reaction mixture, which then turned violet, was heated to 80 °C for 3 h. The mixture was then cooled to room temperature, decanted and filtered and the filtrate concentrated (5 mL) and cooled to −30 °C to give purple microcrystals of the title complex which were isolated by filtration. ^1^H and ^13^C{^1^H} NMR spectra were recorded on a Varian Mercury FT-400 spectrometer and referenced to the residual deuterated solvent. Microanalyses were carried out with a Perkin-Elmer 2400 microanalyzer. IR spectra (KBr pellets were prepared in a nitrogen-filled glove box) were recorded on a Nicolet Avatar 380 FTIR spectrometer (Thermo Fisher Scientific Company, Waltham, MA, USA) in the range 400–4000 cm^−1^.

### 3.3. General Conditions for the Characterization of the Materials

^1^H, ^13^C-CP MAS, and ^29^Si MAS NMR spectra were recorded on a Varian-Infinity Plus spectrometer (Varian Inc., Palo Alto, CA, USA) at 400 MHz operating at a 100.52 MHz proton frequency (4 μs 90° pulse, 4000 transients, spinning speed of 6 MHz, contact time 3 ms, pulse delay 1.5 s). X-ray diffraction (XRD) patterns of the silicas were obtained on a Philips diffractometer (Philips, Amsterdam, The Netherlands), model PW3040/00 X’Pert MPD/MRD, at 45 KV and 40 mA, using Cu Kα with a wavelength of λ = 1.5418 Å. Ti and S wt % determination by X-ray fluorescence were carried out with an X-ray Phillips MagiX fluorescence spectrophotometer (Philips, Amsterdam, The Netherlands) with an X-ray source of 1 kW and a Rh anode in a helium atmosphere. The quantification method is able to analyze from 0.0001% to 100% titanium and sulfur. The thermal stability of the modified mesoporous silicas was studied using a Setsys 18 A (Setaram, Caluire, France) thermogravimetric analyzer, using a 100 μL platinum crucible. A synthetic air atmosphere was used and the temperature was increased from 25 °C to 800 °C at a speed of 10 °C per minute. N_2_ gas adsorption-desorption isotherms were performed using a Micromeritics TriStar 3000 analyzer (Micromeritics, Norcross, GA, USA). Scanning electron micrographs and morphological analysis were carried out on a Philips XL30 ESEM (Philips, Amsterdam, The Netherlands) with an energy dispersive spectrometry system (EDS). The samples were treated with a sputtering method with the following parameters: sputter time 100 s, sputter current 30 mA, film thickness 20 nm using a BAL-TEC SCD 005 sputter coater (Capovani, New York, NY, USA). Conventional transmission electron microscopy (TEM) was carried out on a Philips TECNAI 20 (Philips, Amsterdam, The Netherlands), operating at 200 kV.

### 3.4. Synthesis of SBA-15

The synthesis of SBA-15 was carried out using the experimental procedure reported by Zhao et al. [41]. In a typical synthesis, TEOS 98% aqueous solution (102 g, 0.480 mol) was added dropwise to a stirring solution containing the Pluronic 123 surfactant (48.4 g), 360 mL of Milli-Q water, and 1342 mL of HCl 2 M at 35 °C and 1000 rpm. After the TEOS addition, the reaction was stirred (1000 rpm) at 35 °C for 20 h. During this time, a white solid was formed in the reaction solution. Stirring was then stopped and the temperature was increased to 80 °C and maintained for 24 h in order to complete the ageing process. Afterwards, the solution was filtered under vacuum and the resulting white solid abundantly washed with Milli-Q water to remove soluble impurities and the remaining surfactant. After washing, a drying process (at 100 °C during 6 h) and a subsequent calcination process (during 24 h at 500 °C) were carried out in a muffle oven. After the calcination process, 27.25 g of a fine white powder of SBA-15 was obtained.

### 3.5. Synthesis of SBA-15/[Ti(η^5^-C_5_H_5_)_2_Cl_2_] (S1)

A solution of [Ti(η^5^-C_5_H_5_)_2_Cl_2_] (**1**) (391 mg, 1.57 mmol) (to obtain a theoretical level of 7% Ti/SiO_2_) in toluene (100 mL) was added to dehydroxylated SBA-15 (1.00 g) and the mixture was stirred overnight at 110 °C. The slurry was filtered through fritted discs and the solid residue washed with toluene (5 × 200 mL). The resultant solid was dried under vacuum at room temperature for 16 h to give a light brown free flowing powder.

### 3.6. Synthesis of SBA-15/[Ti(η^5^-C_5_H_5_)(η^5^-C_5_H_4_Pr^i^)Cl_2_] (S2)

The synthesis of material **S2** was carried out in identical manner to that of **S1**. [Ti(η^5^-C_5_H_5_)(η^5^-C_5_H_4_Pr^i^)Cl_2_] (**2**) (458 mg, 1.57 mmol) and dehydroxylated SBA-15 (1.00 g). 

### 3.7. Synthesis of SBA-15/[Ti(η^5^-C_5_H_5_)(η^5^-C_5_H_4_SiMe_3_)Cl_2_] (S3)

The synthesis of material **S3** was carried out in identical manner to that of **S1**. [Ti(η^5^-C_5_H_5_)(η^5^-C_5_H_4_SiMe_3_)Cl_2_] (**S3**) (505 mg, 1.57 mmol) and dehydroxylated SBA-15 (1.00 g).

### 3.8. Synthesis of SBA-15/[Ti(η^5^-C_5_H_5_)_2_{SCH_2_CH_2_CH_2_Si(OEt)_3_}_2_] (S4)

The synthesis of material **S4** was carried out in identical manner to that of **S1**. [Ti(η^5^-C_5_H_5_)_2_{SCH_2_CH_2_CH_2_Si(OEt)_3_}_2_] (**S4**) (1.03 g, 1.57 mmol) and dehydroxylated SBA-15 (1.00 g).

### 3.9. Ti-Release Studies

The titanium release of the materials in biological conditions was carried out in a body simulated fluid. This consists of a pH 7.4 buffer prepared according to previously reported procedures [42]. In duplicate, 20 mL of simulated body fluid was added to 225 mg of the studied materials. These suspensions were incubated at 37 °C in a water bath for 1, 6, 24, 48, and 96 h. Afterwards, the suspension was centrifuged at 2000 rpm during 5 min and subsequently filtered. The solid was washed with Milli-Q water (3 × 5 mL) and dried at 80 °C during 16 h, to eliminate the adsorbed water. The solid was then analyzed by X-ray fluorescence.

### 3.10. DNA-Binding Studies

Fish sperm-deoxyribonucleic acid (FS-DNA) was purchased from Aldrich (St. Louis, MO, USA). The stock solution of FS-DNA was prepared by dissolving an appropriate amount of FS-DNA in tris buffer (pH = 7.4) and storing at 4 °C in the dark. The concentration of the DNA stock solution (2.4 × 10^−4^ M) was determined from the UV absorption spectrum at 260 nm using the molar absorption coefficient ε_260_ = 6600 M^−1^·cm^−1^. The purity of the DNA was checked by the measurement of the ratio of the absorbance at 260 nm to that at 280 nm. The resulting ratio indicated that the DNA was sufficiently free from protein [43,44]. UV absorption spectroscopy experiments were conducted by adding different concentrations of DNA 0.02–0.08 mol/L (nucleotide) to suspensions of **S1**–**S4** (0.5 mg/mL in a mixture ethanol/Tris buffer). The final suspensions were shaken during 30 min at 35 °C and used immediately for the measurements. The changes in the absorbance observed in the spectra were not due to the experimental error, because baseline corrections were applied for all measurements. All measurements were performed at room temperature with an Analytik Jena Specord 200 spectrophotometer (Analytik Jena, Jena, Germany) between 200 and 600 nm.

### 3.11. Biological Studies

The testing was performed on three hepatic, three colon, and two normal human cell lines in vitro. The HepG2 hepatocyte carcinoma, DLD-1 colon adenocarcinoma, HT-29 and COLO320 colorectal adenocarcinoma were obtained from the European Collection of Authenticated Cell Cultures (ECACC), through the Sigma Aldrich Company, St. Louis, MO, USA. The BJ (CRL-2522) normal skin fibroblast cell line was acquired from the American Type Culture Collection (ATCC, Manasses, VA, USA). The Hep3B hepatocellular carcinoma and the SK-Hep-1 human hepatic adenocarcinoma cell line were a generous gift from Dr. Ciprian Tomuleasa from the University of Medicine and Pharmacy “Iuliu Hatieganu”; the cells provenance is the ATCC. The LIV human normal hepatic cells were isolated, characterized [45] and deposited previously by Dr. Ciprian Tomuleasa to *PIO: Platform for interdisciplinary research in oncology* of the The Oncology Institute “I. Chiricuta”. 

Four cell lines presented genetic mutations, according to ATCC (source: ATCC^®^ CELL LINES BY Gene Mutation [46]), being aggressive, highly proliferative tumor cells. SK-Hep-1 display the BRAF and CDKN2A mutations; the COLO320 cells has the APC and TP-53 mutations; DLD-1 is KRAS-, APC-, TP53- and PIK3CA-mutant, while HT-29 displays five major mutations with significance in the evolution of the disease: APC, TP53, PIK3CA, BRAF and SMAD4. 

The cell culture media were: Eagle’s MEM for HepB3, SK-Hep-1, HepG2 and BJ; RPMI-1640 for DLD-1 and COLO320, McCoy’s 5 for HT-29 and a 1:1 mixture of high-glucose DMEM:Ham’s F-12 for LIV cells. All media were supplemented with 10% fetal calf serum and for HepG2 and LIV as well with 1% non-essential amino acids solution. The cells were proliferated on cell culture flasks (Nunclon Easy Flask from Nunc, through Thermo Fisher Scientific Company, Waltham, MA, USA) at 37 °C and 5% CO_2_ and harvested at 70–80% confluence using 0.25% Trypsin-0.053 mM EDTA solution. The only exception was COLO320, which is non-adherent and can be removed from the plate without enzyme. All media, supplements, and reagents were purchased from Sigma Aldrich. 

For viability testing, the cells were plated on 96-well microplates, 1.5 × 10^4^ cells/200 μL per well, while, for Elisa testing, the cells were seeded on 12-well plates, 1.2 × 10^5^ cells/1500 μL. The cells treatment begun at 24 h after the plating, to ensure a good adherence and the commencement of proliferation.

For in vitro testing, **S1**–**S4** were grinded in a glass mortar, and dissolved in phosphate-buffered saline solution (PBS, from Sigma Aldrich), to obtain stock solutions (stable suspensions) of 10 mg/mL. Serial dilutions were prepared in the 1–10,000 μg/mL concentration range. As reference, we used untreated cells, and as blank, PBS solution. The cells treatment was made with a proportion of 1:20 active material in cell culture media: 10:200 μL in the 96-well plates, and 75:1500 μL in the 12-well plates. As positive control, the cytotoxic drug oxaliplatin was used (Oxaliplatin 5 mg/mL, from Actavis Group PTC, Hafnarfiroi, Iceland).

To evaluate the cell growth inhibition MTT (from Sigma Aldrich) colorimetric testing was used; three independent measurements were made, in triplicate. The inhibitory effect against the non-adherent COLO320 cells was measured with the water-soluble MTS dye to avoid repeated centrifugations of the cell culture plate; the reagent was CellTiter 96 aqueous one solution cell proliferation assay, from Promega, Madison, WI, USA. The proliferating capacity was assessed using Alamar blue dye, by fluorescence, as described before [47]. Alamar Blue is a resazurin-based reagent from Invitrogen, acquired through Thermo Fisher Scientific, Waltham, MA, USA.

The soluble interleukin-1 alfa (IL-1α) and tumor necrosis factor receptor 1A (TNFR1) was determined quantitatively by the ELISA method, using kits from R&D Systems, Minneapolis, MN, USA. To quantify the tumor necrosis factor alpha (TNF-α) secreted by the cells, we employed the TNF-alfa Human ELISA kit from Hycult Biotech, Uden, Netherlands, and the procedures were completed following the manufacturer’s instructions.

## 4. Conclusions

Different titanocene derivatives have been immobilized onto nanostructured SBA-15 using simple grafting via protonolysis of the Ti-Cl bond to give SBA-15/[Ti(η^5^-C_5_H_5_)_2_Cl_2_] (**S1**), SBA-15/[Ti(η^5^-C_5_H_5_)(η^5^-C_5_H_4_Pr^i^)Cl_2_] (**S2**), and SBA-15/[Ti(η^5^-C_5_H_5_)(η^5^-C_5_H_4_SiMe_3_)Cl_2_] (**S3**). In addition, a tethering reaction of a titanocene derivative containing triethoxysilyl fragments [Ti(η^5^-C_5_H_5_)_2_{SCH_2_CH_2_CH_2_Si(OEt)_3_}_2_] (**4**) with SBA-15 was carried out to give the material SBA-15/[Ti(η^5^-C_5_H_5_)_2_{SCH_2_CH_2_CH_2_Si(OEt)_3_}_2_] (**S4**). All materials were characterized by different methods observing the incorporation of the titanocene derivatives mainly inside the pores of the SBA-15-based systems. **S1**–**S3** showed lower titanium content than **S4** confirming that the functionalization rates are higher when using ligands with triethoxysilyl fragments. The cytotoxicity of the materials **S1**–**S4** was evaluated on different hepatic and colorectal cell lines observing that in all cell lines, **S3** and **S4** showed higher cytotoxicity than **S1** and **S2** and better selectivity than oxaliplatin. 

HT-29 and DLD-1 colon cell populations, both bearing the APC, TP53, and PIK3CA mutations, are indicators of poor prognosis in colorectal pathology. Therefore, these promising results regarding the in vitro cytotoxicity of **S4** may be a premise for further studies on this material. In addition, **S3** and **S4** are able to induce cell growth inhibition by interfering with the metabolic activity of the cell and through TNFR1 modulation, a phenomenon that has not been described until now for metallodrug-functionalized nanostructured systems.

## Figures and Tables

**Table 1:** Percentage of Ti grafted on the silica support.

Material	Theoretical wt % Ti	Experimental wt % Ti ^1^	Experimental mmol of Ti/g Material
**S1**	7	1.41	0.869
**S2**	7	1.32	0.869
**S3**	7	1.33	0.869
**S4**	7	6.47	1.739

^1^ Determined by X-ray fluorescence.

**Table 2:** XRD (X-ray diffraction) data of SBA-15, **S1**–**S4**.

Material	SBA-15		S1		S2		S3		S4	
**Miller index**	100	110	100	110	100	110	100	110	100	110
**Interplanar distance (Å)**	73.59	14.77	74.33	14.83	72.09	14.71	73.37	14.71	74.56	14.94
**2θ (°)**	0.93	1.84	0.92	1.83	0.95	1.85	0.93	1.83	0.91	1.81

**Table 3:** Physical parameters of SBA-15, **S1**–**S4** measured by N_2_ adsorption-desorption isotherms.

Materials	S_BET_	Total Pore Volume	D_P_ (BJH) ^1^	*d*_100_	Wall Thickness ^2^	V_P_
	(m^2^/g)	(cm^3^/g)	(Å)	(Å)	(Å)	(cm^3^/g)
**SBA-15**	857	0.88	71.5	73.6	13.5	0.88
**S1**	792	0.83	69.8	74.33	16.02	0.83
**S2**	850	0.90	42.1	72.09	41.14	0.90
**S3**	793	0.85	42.9	73.37	41.82	0.85
**S4**	520	0.59	46.1	74.56	39.99	0.59

^1^ Barrett, Joyner and Halenda; ^2^ Wall thickness was calculated using the following equation: Wall thickness = $\frac{2d_{100}}{\sqrt{3}}$ − BJH average pore diameter.

**Table 4:** Half maximal effective concentrations (EC_50_) of the mesoporous nanoparticles **S1**–**S4** against human tumor and normal cells in vitro; median values obtained from three independent measurements; SD represent the standard deviation in the 95% confidence interval.

Cell Line	EC_50_ [μg/mL]				
	Material/Compound				
	S1	S2	S3	S4	Oxaliplatin
**HepG2**	613.3 ± 29.8	769.7 ± 25.7	556.8 ± 40.9	403.3 ± 20.8	338.9 ± 11.6
**Hep3B**	298.0 ±15.7	145.3 ± 27.2	78.9 ± 4.2	66.9 ± 3.9	96.1 ± 12.4
**SK-Hep-1**	235.3 ± 26.2	272.7 ± 38.4	95.5 ± 8.9	79.5± 9.3	68.3 ± 8.0
**DLD-1**	438.6 ± 39.1	227.4 ± 16.2	157.5 ± 25.7	94.2 ± 10.3	142.5 ± 45.9 [33]
**HT-29**	155.3 ± 18.4	165.7 ± 12.3	129.1 ± 10.8	85.6 ± 5.8	209.0 ± 13.2 [33]
**COLO320**	662.0 ± 21.2	470.8 ± 34.9	306.8 ± 9.7	393.4 ± 12.7	88.6 ± 0.5
**LIV**	757.2 ± 193.2	>1000	>1000	>1000	217.1± 6.9
**BJ**	>1000	>1000	958.0 ± 168.8	658.5 ± 231.6	252.4 ± 11.8

**Table 5:** Influence of **S1**–**S4** on the metabolic activity of the tumor cell populations, expressed as linear regression of the changes in reducing potential within a 24-h exposure. The deviation of the slope from 0 is an indicator of the cellular metabolic decline, and it is significant when *p* < 0.05.

Cell Line	Deviation from 0	Material S1	Material S2	Material S3	Material S4
**HepG2**	Hillslope	−749.6 ± 481.2	−352.2 ± 292.4	−534.3 ± 425.7	−1066 ± 328.2
	*p* value	0.1416	0.2483	0.2299	0.0058
**Hep3B**	Hillslope	−1048 ± 145.2	−1267 ± 23.2	−1436 ± 167.0	−1462 ± 185.3
	*p* value	< 0.0001	< 0.0001	< 0.0001	0.0002
**SK-Hep-1**	Hillslope	−1215 ± 236.1	−1326 ± 234.2	−1876 ± 358.2	−1116 ± 308.6
	*p* value	0.0001	< 0.0001	0.0001	0.0028
**DLD-1**	Hillslope	−360.9 ± 209.1	−719.3 ± 129.1	−916.2 ± 216.6	−1400 ± 319.7
	*p* value	0.0610	0.0063	0.0106	0.0005
**HT-29**	Hillslope	−1082 ± 278.3	−1066 ± 286.3	−2063 ± 277.8	−1263 ± 212.1
	*p* value	0.0013	0.0019	< 0.0001	< 0.0001
**COLO320**	Hillslope	−369.0 ± 227.8	−278.8 ± 188.4	−683.7 ± 112.0	−1195 ± 236.5
	*p* value	0.1276	0.1612	< 0.0001	0.0002

**Table 6:** Release studies of material **S1** in simulated body fluid.

t (h)	wt % Ti in S1	Release (%) in S1	wt % Ti in S4	Release (%) in S4
**0**	1.26	0	6.47	0
**1**	1.12	11	6.10	6
**6**	1.14	9	5.83	10
**24**	1.18	6	5.12	21
**48**	1.21	4	4.77	26
**96**	1.19	5	4.48	31

## Supplementary Materials

The following are available online at http://www.mdpi.com/1996-1944/11/2/224/s1, Figure S1. Pore size distributions of the all the synthesized materials where a homogeneous narrow distribution is observed; Figure S2. Thermogravimetric analysis of materials SBA-15, **S1**–**S4**; Figure S3. UV-VIS spectra of titanocene dichloride, SBA-15, and **S1**–**S4**; Figure S4. FTIR spectra of titanocene dichloride, SBA-15, and **S1**–**S4**.
