# Supplementary material for: Anticancer Applications of Nanostructured Silica-Based Materials Functionalized with Titanocene Derivatives: Induction of Cell Death Mechanism through TNFR1 Modulation
Source: Materials (Basel). 2018 Jan 31;11(2):224. doi: 10.3390/ma11020224 (PMC5848921; doi:10.3390/ma11020224)
Supplement: Supplementary file 1 [file materials-11-00224-s001.pdf]

materials

Supplementary Material

Anticancer Applications of Nanostructured Silica-Based Materials Functionalized with Titanocene Derivatives: Induction of Cell Death Mechanism through TNFR1 Modulation

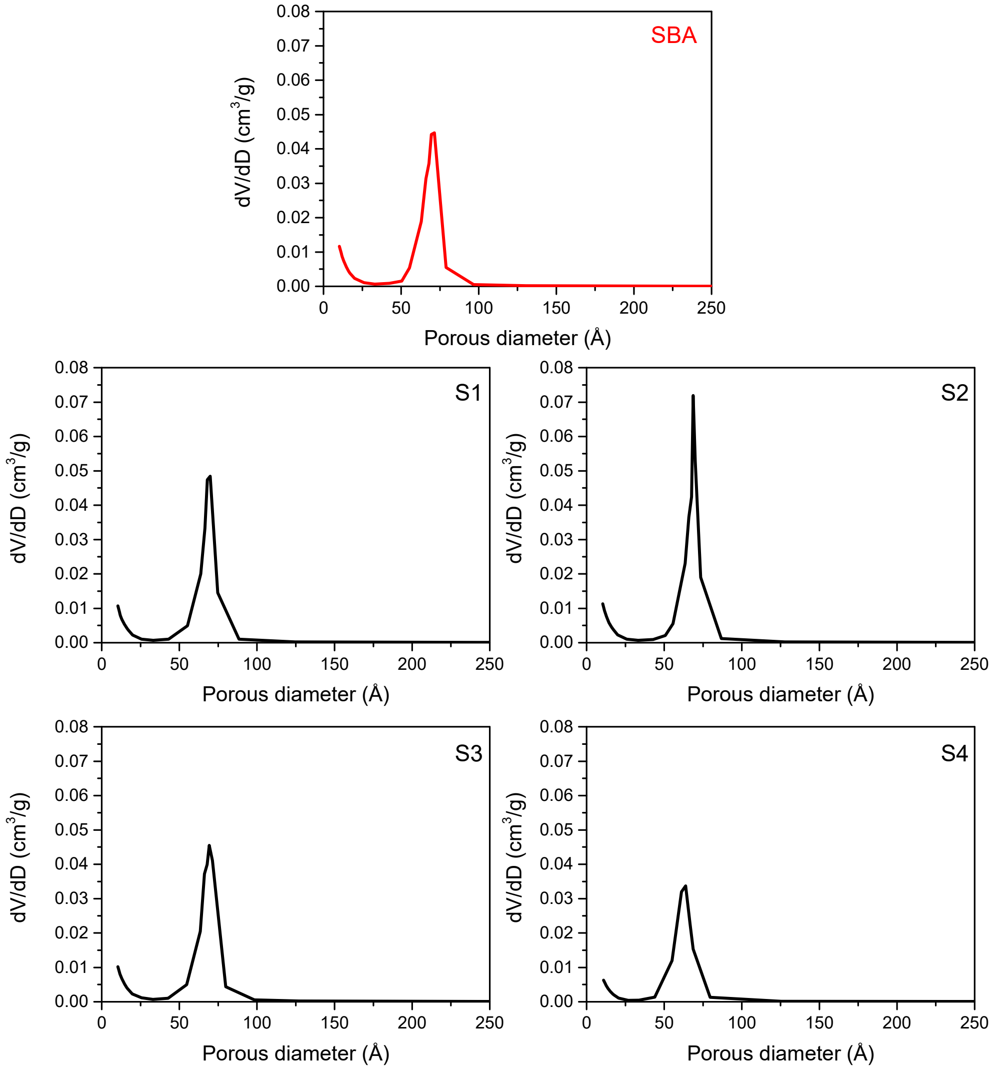

Figure S1. Pore size distributions of the all the synthesized materials where a homogeneous narrow distribution is observed.

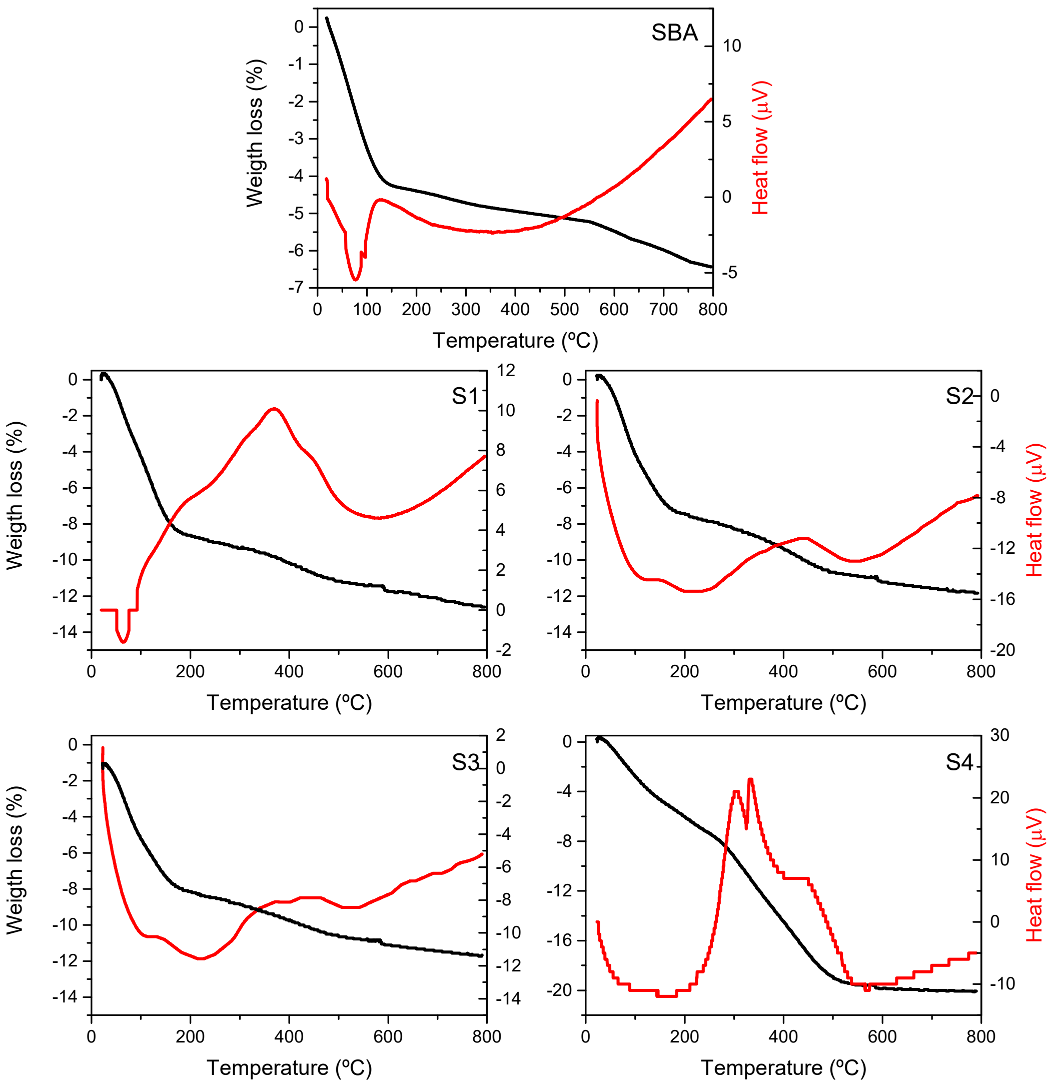

Figure S2. Thermogravimetric analysis of materials SBA-15, **S1**, **S2**, **S3** and **S4.**

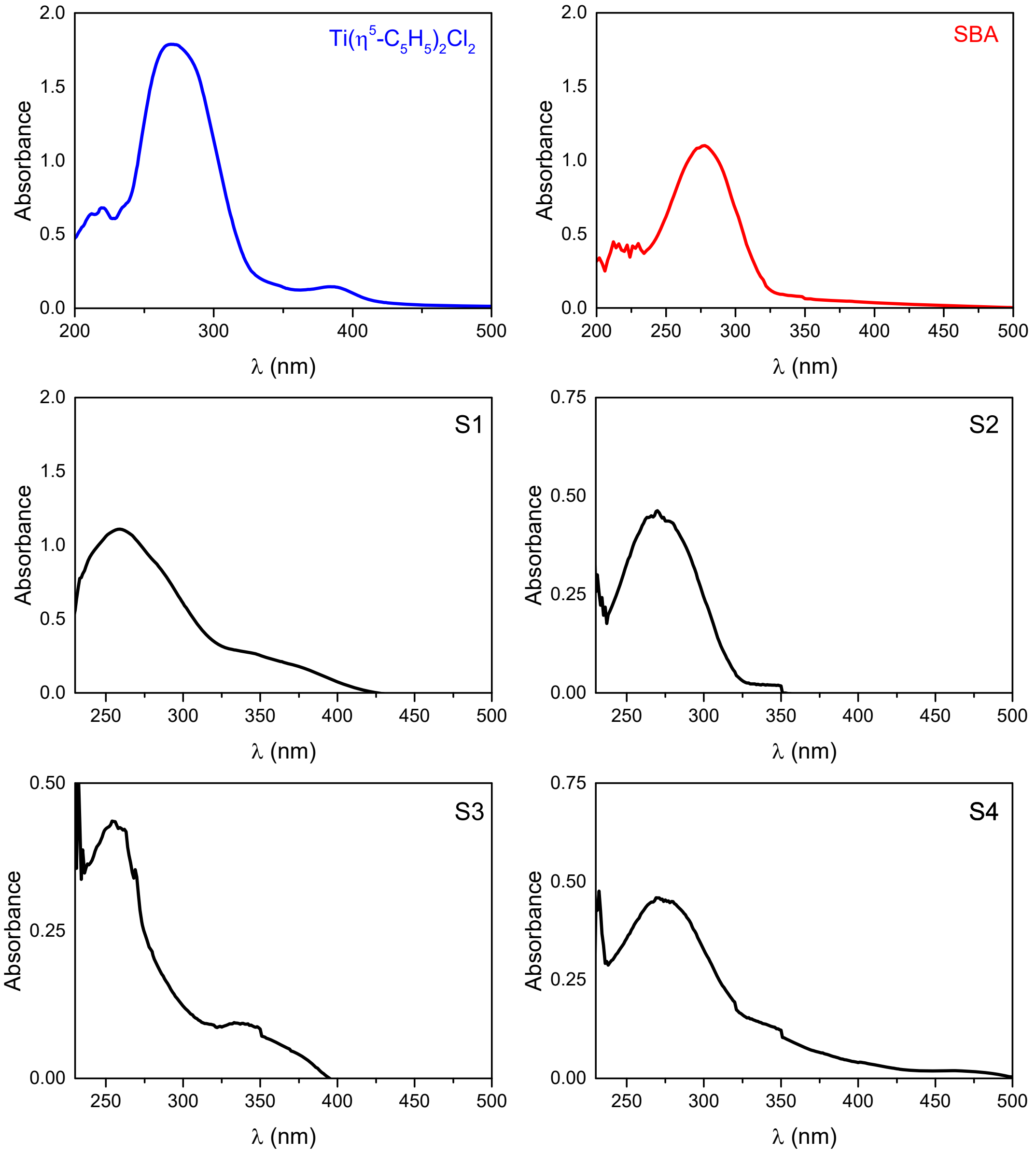

Figure S3. UV-vis spectra of titanocene dichloride, unmodified SBA-15 and **S1–S4**.

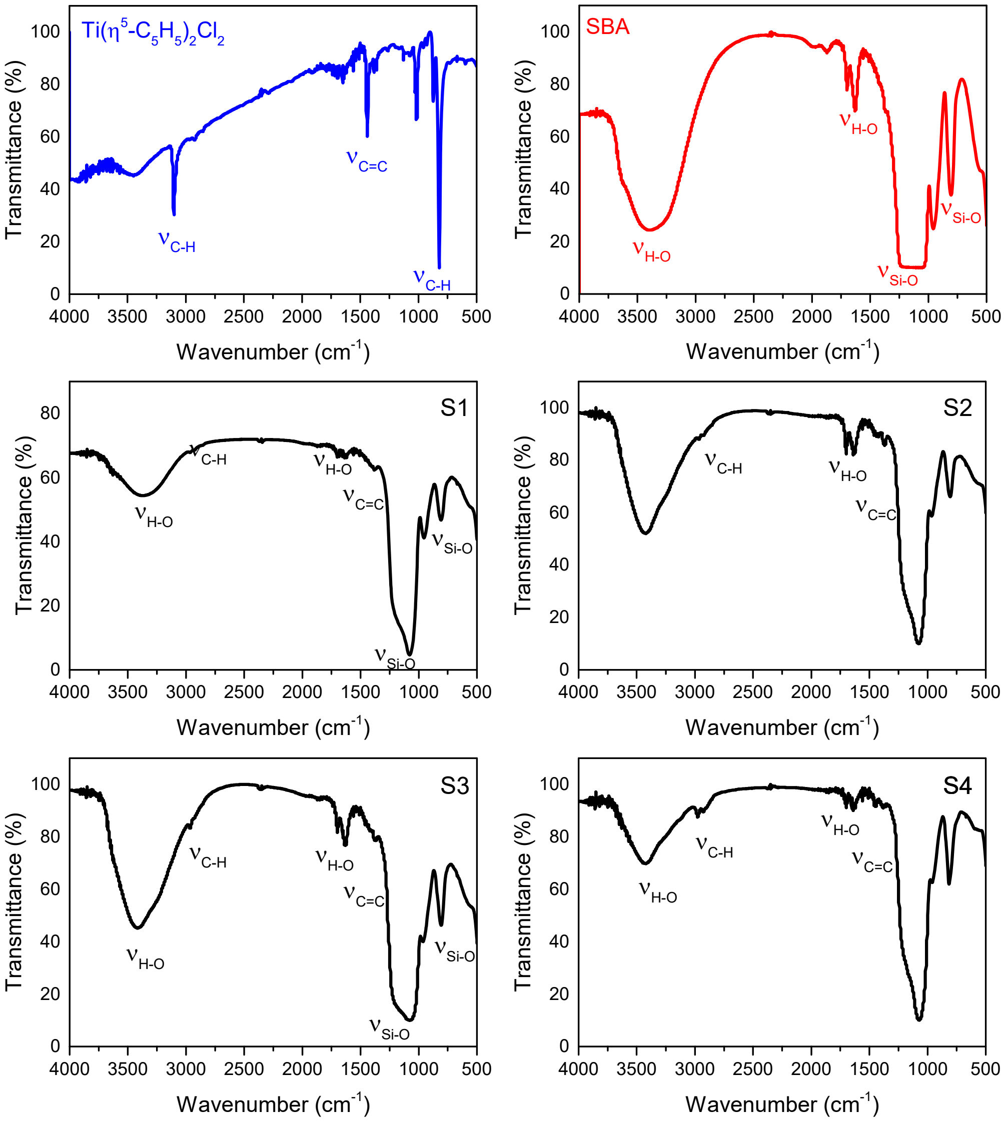

Figure S4. FT-IR spectra of titanocene dichloride, unmodified SBA-15 and **S1–S4**.
